# Anterior Urethral Laceration from a Human Bite

**DOI:** 10.5811/cpcem.2017.6.34323

**Published:** 2017-10-03

**Authors:** Chadwick Shirk, Wesley Eilbert

**Affiliations:** University of Illinois at Chicago, College of Medicine, Department of Emergency Medicine, Chicago, Illinois

## Abstract

Isolated anterior urethral injuries in males related to sexual activity have rarely been reported. Human bites to the penis are also rarely discussed in the medical literature. We report an isolated anterior urethral laceration in a male caused by a biting injury sustained during fellatio.

## INTRODUCTION

Most reported male genital injuries related to sexual activity have been penile fractures, with rupture of the corpora cavernosa often due to vigorous or alternative positions during coitus.^1^ Associated urethral injury with penile fracture occurs in 10–38% of cases.^2^ Isolated male urethral injury due to sexual activity is exceptionally rare 3–6 and has never been reported as having been caused by fellatio.

Most of the published literature on bite injuries of the male genitalia has involved animal bites.^7^–^9^ Human bite injuries to the penis is a topic rarely discussed in the medical literature. These injuries are probably underreported because of embarrassment,^10^,^11^ and there is frequently a delay in seeking treatment.^11^ To date, only infectious complications and amputation from human bites to the penis have been reported.^11^–^14^

## CASE REPORT

A 30-year-old male with no significant past medical history presented to the emergency department (ED) complaining of a bite wound to his penis that had occurred approximately one hour prior to arrival. The bite occurred while receiving oral sex from his girlfriend, and he was unsure if it was intentional or accidental. He stated there was some bleeding from the wound that stopped with direct pressure. He had not urinated since the injury. Physical examination revealed a 0.5 cm superficial skin avulsion on the ventral aspect of the mid-penile shaft in the midline. Several other superficial excoriations were noted on the penile shaft. A small amount of blood was noted at the urethral meatus. The patient was able to urinate, and urinalysis revealed >100 red blood cells per high power field (hpf) and 5–10 white blood cells/hpf.

Because of concern for an anterior urethral injury, a retrograde urethrogram was performed (Image). The lumen of the midportion of the penile urethra was noted to be irregular with a small amount of contrast extravasation indicative of partial laceration of the urethra. A 16 gauge Foley catheter was placed without difficulty, and the patient was discharged with a leg bag and a prescription for seven days of prophylactic antibiotics. Due to a lack of medical insurance, the patient was unable to follow up with a urologist, and he returned to the ED six days later. His Foley catheter was removed at that time and he was able to void without difficulty.

## DISCUSSION

Bite injuries to the penis are rarely reported. A study of human bites reported to the New York City Department of Health found that of 892 human bites, only two (0.2%) were to the penis.^15^ In a study of traumatic penile injuries, 85% were due to a blunt mechanism, with all of these blunt injuries occurring during either sexual intercourse or masturbation.^16^ In another study specifically examining urethral injuries from blunt penile trauma, 91% of cases occurred during sexual intercourse and all had associated corporal injury.^1^ To our knowledge, isolated urethral injury from a human bite has never been reported.

Blood at the urethral meatus after blunt penile trauma is the cardinal sign of anterior urethral injury, though it is only 75% sensitive.^17^ Other clinical signs include dysuria, hematuria and inability to void.^18^ Delays in diagnosis with urinary extravasation into the surrounding tissues may result in severe and necrotizing local infection as well as sepsis.^18^

A retrograde urethrogram remains the gold standard for diagnosing urethral injury.^19^ Retrograde urethrography can distinguish between complete transection and partial laceration, as seen with our patient. With partial urethral laceration, there is extravasation of contrast into the periurethral soft tissues with continued filling of the urethra proximally. With complete transection, contrast doesn’t progress proximal to the area of extravasation.

Management of complete urethral transections involves suprapubic urinary diversion by placement of a suprapubic catheter. This can usually be performed percutaneously in the ED, typically by the Seldinger technique. Delayed primary repair of the urethra can then be performed at a later date.^17^ Management of partial urethral laceration is more controversial. Traditionally, catheterization of partial tears was discouraged to prevent the potential conversion of a partial into a complete urethral injury. Little evidence exists to support this risk of conversion, and one gentle attempt to place a Foley catheter in a partial disruption is reasonable.^17^ If successful, the Foley catheter should remain in place for one to two weeks to allow adequate time for the urethra to heal.^17^

As with our patient, all human bites with associated injury to underlying structures should receive prophylactic antibiotics. Amoxicillin-clavulanate is the antibiotic of choice for this purpose.^20^

**CPC-EM Capsule**

What do we already know about this clinical entity?

Human bites to the penis are rarely reported in the medical literature, and isolated urethral injury from a human bite has never been reported.

What makes this presentation of disease reportable?

There have been no prior reports of injury to the male urethra from a human bite.

What is the major learning point?

Human bites to the penis may result in significant urethral injury, even in the absence of major external damage.

How might this improve emergency medicine practice?

Recognition and treatment of potential urethral injuries from human bites will prevent associated long-term complications.

## CONCLUSION

Human bites to the penis are rarely discussed in the medical literature. To our knowledge, this is the first reported case of urethral laceration caused by a human bite. Urethral injury should be considered with all penile bite injuries, no matter how innocuous the surface wound appears.

## Figures and Tables

**Image:**
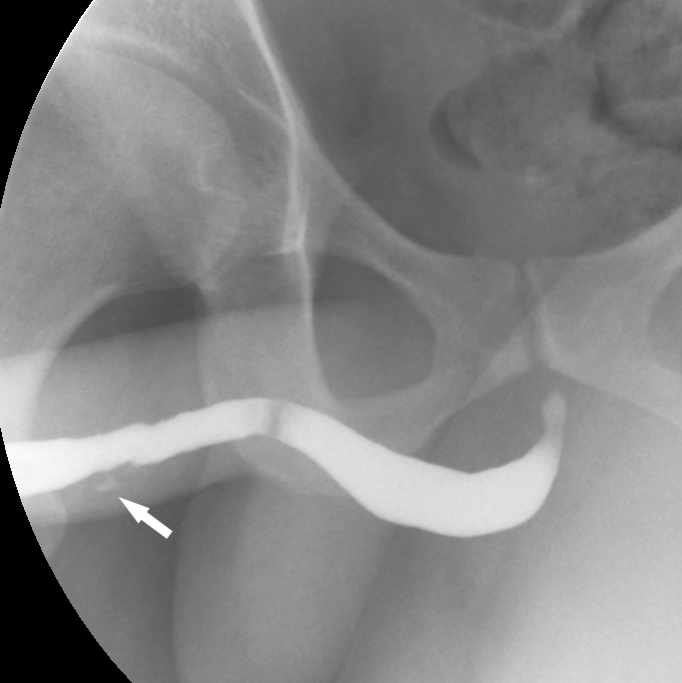
Retrograde urethrogram demonstrating an irregular lumen of the midportion of the penile urethra with a small amount of contrast extravasation (arrow).
